# C.R.A.B.: a gamified paradigm for studying readiness potential

**DOI:** 10.3389/fnhum.2025.1534412

**Published:** 2025-09-03

**Authors:** Evgeny Blagovechtchenski, Maria Koriakina, Ksenia Bartseva, Alexandra Kuznetsova, Aleksandr Kirsanov, Sofia Ponomareva, Alena Popyvanova, Ekaterina Pomelova

**Affiliations:** ^1^Laboratory of Behavioural Neurodynamics, St. Petersburg State University, St. Petersburg, Russia; ^2^Centre for Cognition and Decision Making, National Research University Higher School of Economics, Moscow, Russia

**Keywords:** free will, EEG, ERP, experimental design, game

## Abstract

**Background:**

This study presents a novel paradigm termed Contrasting Routines Affecting Bereitschaftspotential (C.R.A.B.), designed to analyze readiness potential (RP) waveforms preceding movements across varying experimental settings. This paradigm continues Libet's work on decision-making, with an emphasis on the motor planning component like in classical Libet experiment. However, existing paradigms for studying RP work poorly across ages (requiring different instructions) and do not accurately identify the components associated with RP formation.

**New method:**

The C.R.A.B. paradigm enables modeling the when-decision through indirect measurements, thereby avoiding reliance on participants' introspective reports. We believe that this framework can isolate the motor planning component from decision-making and is also suitable for implementation with children of different ages and disorders.

**Results:**

As a proof of concept, we employed the C.R.A.B. paradigm with healthy adult participants to validate its effectiveness. Our findings revealed distinct RP waveform variations across different trial conditions. Comparison with existing methods C.R.A.B. paradigm has several advantages over traditional methods: the design of the experiment provides for the assessment of different components of RP—motor planning, attention level, and spontaneity of decision-making since the design of the experiment is developed in a game form, it is suitable for both children and adults.

**Conclusions:**

The C.R.A.B. paradigm effectively demonstrated differences in RP waveforms across trial types; therefore, this paradigm can be used to study the various components of the RP in detail. Since this paradigm represents a game interface, it is possible to study the RP in children, including children with various disorders.

## Introduction

The concept of the Readiness Potential (RP) was first documented in 1964 (Deecke et al., 1969; Kornhuber and Deecke, 1990) and is also referred to as the Bereitschaftspotential (a German term that translates as “readiness potential”). This phenomenon was initially associated with the activation of the motor cortex and the supplementary motor area of the brain, which are involved in the planning of precise voluntary movements. However, subsequent experiments by (Libet et al. 1983) showed that this potential is also associated with decision-making mechanisms, including unconscious ones. The fundamental question concerns the distinction between motor planning and decision-making processes. Of particular interest is the ability to assess changes in RP with age, especially in children (see below). In this methodological article, we have proposed a gamified paradigm that is suitable for individuals of different ages. Moreover, by modifying the settings of this design, it becomes possible to assess different components of RP in addition to other features of decision making and motor planning.

Studies focusing on premovement motor-related cortical potentials have traditionally focused on adult participants, including Libet's (1983) seminal study. Meanwhile, the developmental perspective on RP and other premovement potentials especially in children remains underexplored. The self-paced movement paradigm has been used in the limited literature aimed at assessing the parameters of RP in children (e.g., Jarczok et al., 2019). To study lateralized readiness potential (LRP) waveforms in an alternative setting, a version of the Stroop task that manipulates the size of the animal (animal-size Stroop task) was used (Bryce et al., 2011; Nayak et al., 2020; Szucs et al., 2009). However, this approach did not aim to track the development of movement preparation over time but instead focused on response competition and inhibitory control mechanisms. In addition, many factors can influence LRP, such as sleep (Song et al., 2023), which is also age dependent.

Another approach was suggested by (Cavazzana et al. 2014), who used a stream of letters, similar to the study by (Soon et al. 2008), to examine intentional binding (the subjective compression of the temporal interval between a voluntary action and its external sensory consequence) in children. Remarkably, no such effect was found in children, unlike in adults (Cavazzana et al., 2014). The authors noted that the traditional Libet's clock (subjective assessment of the moment of decision making), which has also been used to study intentional binding (Haggard et al., 2002), does not seem to be a suitable solution for children due to the difficulties they might face in monitoring the clock's rotation while tracking their introspective sensations. However, one might think that the perception of a stream of letters might also be too demanding for children. This is particularly relevant for younger children, who may not have fully developed reading skills or whose skills are significantly affected by socioeconomic factors, resulting in greater variability in reading skills up to the age of 8–10 years (Suggate et al., 2013) (Supplementary Table 1).

Nevertheless, these approaches did not aim to track the development of RP over time in a Libet-like manner, which would have required participants to make their when-decisions according to the classification by (Brass and Haggard 2008): “a component related to the decision about which action to execute (*what* component), a component that is related to the decision about when to execute an action (*when* component), and finally the decision about whether to execute an action or not (*whether* component).” Our primary motivation was to compare the development of RP waveforms in children, focusing mainly on healthy children and those with different conditions, such as motor disorders, autism, and others (Anguelova et al., 2016; Bahm et al., 2009; Mehta et al., 2006). Given that no appropriate design was found in the literature that satisfied our requirements, we aimed to develop a novel framework to assess the development of premotor electrophysiological activity considering the three types of decisions proposed by (Brass and Haggard 2008): when-decisions, what-decisions, and whether-decisions.

To achieve these goals, we proposed a gamified paradigm, Contrasting Routines Affecting Bereitschaftspotential (C.R.A.B.), that can be used to track the development of pre-movement neuronal activity over time and disentangle various factors related to decision-making that affect the RP waveform. To provide proof of concept for this approach, we conducted a pilot study on healthy adults using a bimanual version of the game. We hypothesized that the three experimental conditions used in this framework would elicit prominent RP waveforms that would differ in amplitude and onset parameters.

## Methods

### C.R.A.B. game

The goal of the game was to create a scenario in which it is possible to separate the moment of decision-making into three paradigms: the correct decision is known in advance; the correct decision is unknown until the last second and becomes clear only at the last moment; and the correct decision is not known a priori—a spontaneous decision. The separation of evoked potentials (EP) in EEG using this method may allow us to distinguish motor planning from the actual process of decision-making.

**Figure 1:**
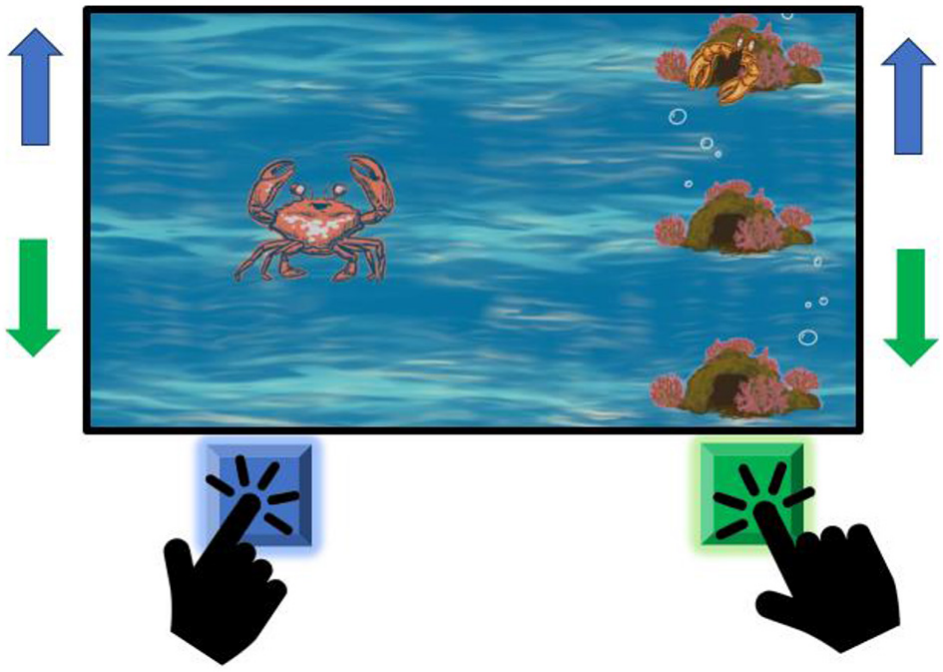
The gameplay of the paradigm. Throughout the trial, the actor (red crab) moves toward the hider (orange crab). The goal of the participant is to make the actor meet the hider.

The game consisted of a series of rounds of hide-and-seek, each of which corresponded to one experimental trial (see the video in Supplementary materials). The participants were instructed to help the avatar (the actor-crab) find one of three hiding game characters (the hider-crabs) by navigating the actor-crab along one of three non-overlapping trails (an upper trail, a middle trail, and a lower trail) (Figure 1). At the beginning of each hide-and-seek round, the actor-crab started moving rightward from its initial position on the middle trail and finished in one of the three hiding spots. If the actor-crab ended up in the hiding spot where the hider-crab was located at the end of the hide-and-seek round, the participant earned 1 point and won that particular round of the game. If the actor-crab finished in a hiding spot with no hider-crab, the participant earned 0 points for that particular round. The duration of the actor-crab's movement from its initial position to one of the hiding spots was 5,000 ms. Importantly, at any point during the hide-and-seek round, the participant could change the trajectory of the actor-crab's movement by shifting it from the middle trail to the upper or lower trail. However, the trajectory could only be changed once per round.

All hide-and-seek rounds were divided into three types corresponding to their difficulty levels: “easy,” “medium,” and “hard.” Each trial type corresponded to a particular hider-crab avatar. We want to emphasize that all game settings and decision-making moments can be edited in the experiment's program code.

In the easy trials, the participants observed the hider-crab's hiding spot from the very beginning of the actor-crab's 5-s walk. Because the hiding spot remained unchanged throughout the round, the participant was aware of the hider-crab's final location from the start, which greatly facilitated decision-making.

In the medium trials, the participant could similarly observe the hider-crab's hiding spot during the actor-crab's 5-s walk. However, the hider-crab could change its hiding spot by “jumping” to a neighboring hiding spot at any time during the actor-crab's walk. The hider-crab could make up to three jumps, with both the number of jumps and their timings chosen randomly before each trial. Therefore, the participant needed to maintain a high level of attention until the very last moment of decision-making.

Finally, in the hard trials, the hider-crab was invisible during the entire period of the actor-crab's walk from the starting spot to one of the hiding spots along the chosen trajectory. Accordingly, as the participants could not use visual information to select the trail followed by the hider-crab, they had to rely on spontaneous decision-making.

Importantly, before the experimental session began, the participant was presented with a short story explaining the fictional narrative of the three hider-crabs, corresponding to the three experimental conditions. The participant observed the game mechanics used in all three types of trials and was subsequently able to identify the forthcoming game type based on the shape and color of the hider-crab at the beginning of each hide-and-seek round.

In the bimanual edition of the game, the left-hand movement corresponded to moving the actor-crab one trail up (from the middle trail to the upper trail), while the right-hand movement corresponded to moving the actor-crab one trail down (from the middle trail to the lower trail). In the unimanual version, the participant used a three-button game controller operated with their dominant hand. The task's difficulty is coded by the color of the crab the subject is manipulating.

Accordingly, the participants implemented all three decision types suggested by (Brass and Haggard 2008). What-decisions were made by choosing upward or downward movement in the bimanual edition or by selecting one of the three buttons on the controller in the unimanual edition. When-decisions were made by choosing the moment to press the button to change the actor-crab's trajectory in the bimanual edition, or to select the hiding spot of interest in the unimanual edition. Whether-decisions were recorded only in the bimanual edition and represented the participant's choice to change the actor-crab's trajectory from the middle trail to the upper or lower trail or to leave it unchanged by taking no action.

**Figure 2:**
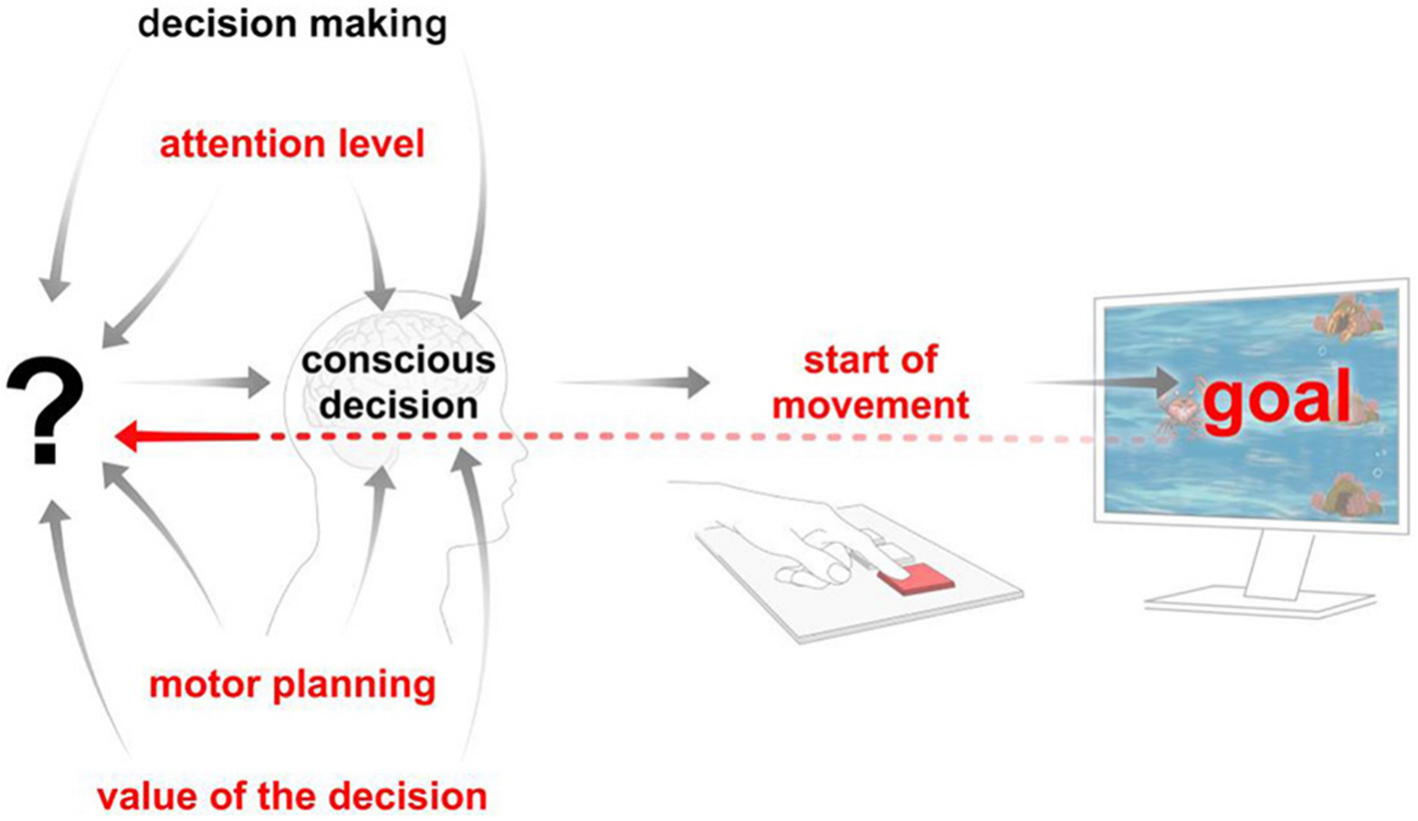
The factors that can be controlled in the proposed paradigm are marked in red, and the way these factors interact is illustrated.

As demonstrated in Figure 2, the paradigm under study differs from other paradigms in the literature (see Supplementary Table 1). The parameters that this game design allows for control are marked in red. The central theme of this study is the analysis of the various components that, when considered as a whole, give rise to the phenomenon of “free will.”

### Software for the C.R.A.B. game

The stimuli were created in Python 3.10 using the PsychoPy library for graphical support and high-precision response registration (Peirce et al., 2019). The open-source code is available in the author's GitHub repository (github.com/DimitriBr).

### Proof-of-concept testing: participants

The pilot study involved a group of 23 healthy adult volunteers (16 females and 7 males). The participants had a mean age of 21.1 years, with a standard deviation of 2.1, and were recruited through social networks and campus advertisements to ensure a diverse sample. Before data collection, all participants received detailed information about the experimental procedure and provided written informed consent. As an incentive for their participation, the volunteers were compensated with an average of 250 rubles per hour, as the study lasted ~2 h. The project received ethical approval from the HSE Committee on Interuniversity Surveys and Ethical Assessment of Empirical Research, ensuring compliance with ethical guidelines and standards.

### Proof-of-concept testing: recording

Electroencephalograms were recorded during the game using a 64-electrode setup (ActiCap, BrainProducts) placed according to the international 10–20 system, with Cz as the reference electrode and the forehead electrode as the ground electrode (Sehatpour and Javitt, 2024). The impedance of all electrodes was kept below 10 k℧. The EEG signal was amplified by a BrainAmp DC (BrainProducts) and recorded using a 0.01–100 Hz bandpass filter and a 50 Hz notch filter. The signal was digitized with a sampling frequency of 500 Hz.

### Proof-of-concept testing: EEG pre-processing

The EEG signal was filtered with a 0.1–40 Hz bandpass. Biological artifacts (blinks and eye movements) were removed using decomposition after independent component analysis (Infomax algorithm). Based on visual inspection, only epochs without excessive noise were retained for further analysis. The remaining trials (at least 30 for each condition) were averaged. The signal-to-noise ratio was subsequently calculated for each participant: the mean amplitude of the 400 ms pre-movement signal (averaged RP peak) was normalized by the standard deviation across the entire pre-movement duration of the epoch. If a participant's signal-to-noise ratio was below 1 in any of the conditions, that participant was excluded from the analysis (*N* = 4).

**Figure 3:**
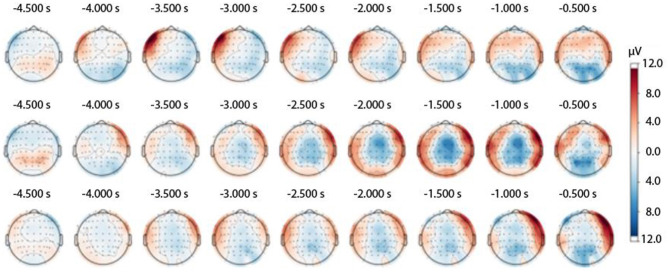
Topography of the RP waveform throughout the experimental session in the three experimental trial types: easy trials **(Upper panel)**, medium trials **(Middle panel)**, and hard trials **(Lower panel)**.

**Figure 4:**
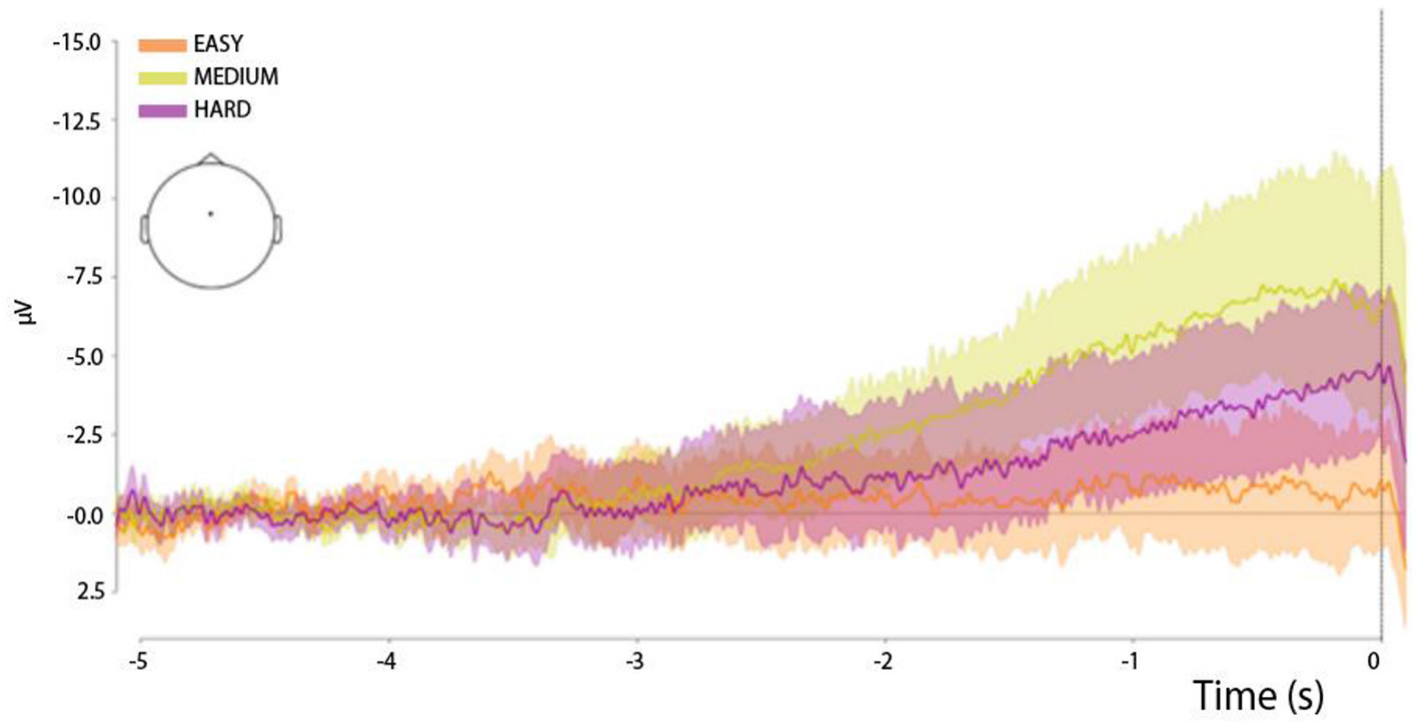
Button-press-locked (0 ms—button press) RP waveforms in the three experimental trial types: easy trials, medium trials, and hard trials. All time series were derived from the Cz channel. The shadows of the curves reflect the spread of the data.

**Figure 5:**
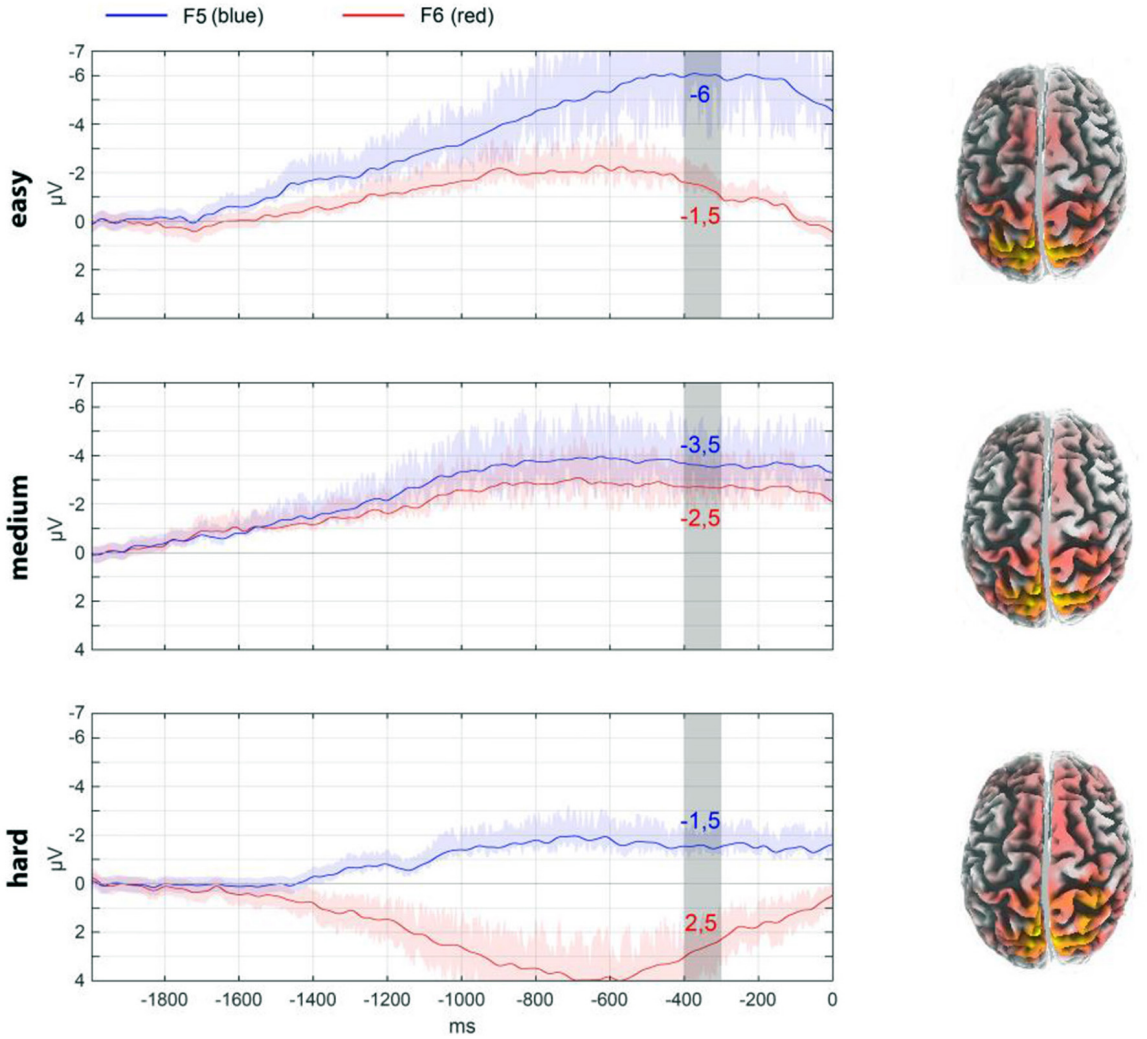
The lateralization of responses under different conditions is illustrated using frontal electrodes F5 and F6 as an example. To ease evaluation, the data are averaged for the interval −400 to −300 ms. The ordinate scale is shown in μV. The source reconstruction for this interval by eLoreta is shown on the right of the panel. The shadows of the curves reflect the spread of the data.

**Figure 6:**
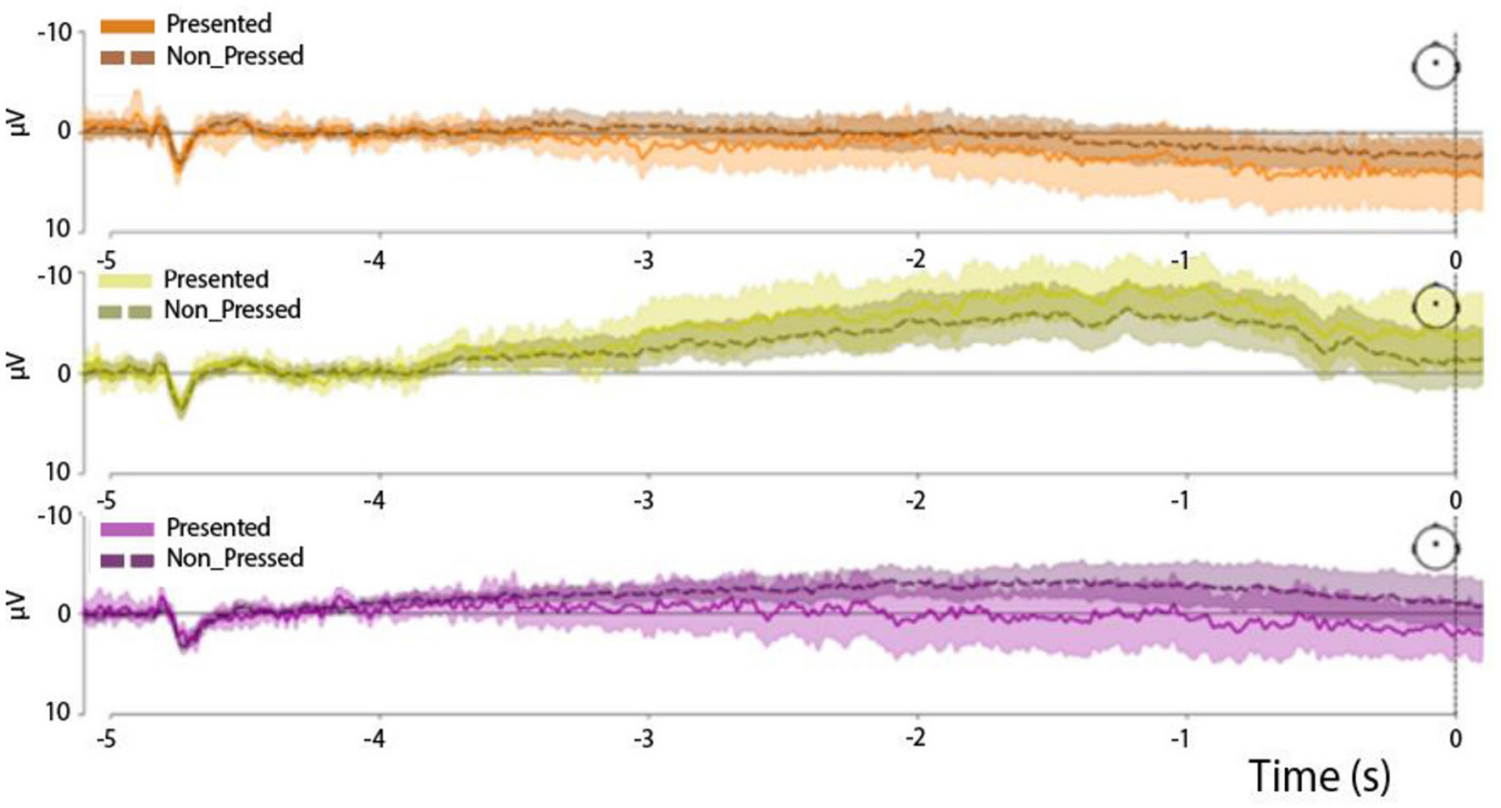
End-of-trial-locked RP waveforms in the three experimental trial types: easy trials **(Upper panel)**, medium trials **(Middle panel)**, and hard trials **(Lower panel)**. The solid line represents the time series terminated by a button press, while the dashed line represents the time series not followed by any button presses. All time series were derived from the Cz channel.

Subsequently, the identified epochs in the EEG were analyzed. In order to construct Figures 3, 4, the baseline was determined to be from −5,000 to −4,800 ms before the button was pressed. For Figure 5, the timeframe was set to −2,100 to −1,900 ms. Finally, for Figure 6, the baseline was set to −5,100 to 4,900 ms. For these figures, EP estimates were made for specific triggers.

The activity sources in the −400 to 300 ms interval were also analyzed. Specifically, an eLORETA solution (Pascual-Marqui et al., 2011) was computed on a realistic head model based on the Montreal Neurological Institute (MNI) template, employing a boundary-element method to account for the conductivity properties of head tissues. Source reconstruction was constrained to cortical gray matter and applied to grand-averaged data to enhance signal-to-noise ratio. The analysis was performed using the sLORETA/eLORETA software package on the mean ERP of dissonant and neutral conditions within the peak time intervals identified in the signal-space ERP analysis (https://www.uzh.ch/keyinst/NewLORETA/Software/Software.htm).

## Results

Behavioral results showed that in the case of Easy Trail the success rate was 100%, in the case of Medium Trail−71% (±9), Hard Trail−49% (±11).

Over the course of this study, we tracked the development of the RP waveform. We confirmed that the typical RP topography was registered in the medium and hard trials (Shibasaki and Hallett, 2006). In contrast, ultra-rapid reactions to stimulus presentation in the easy trials were not accompanied by a typical RP spatial pattern (see Figure 3).

**Figure 7:**
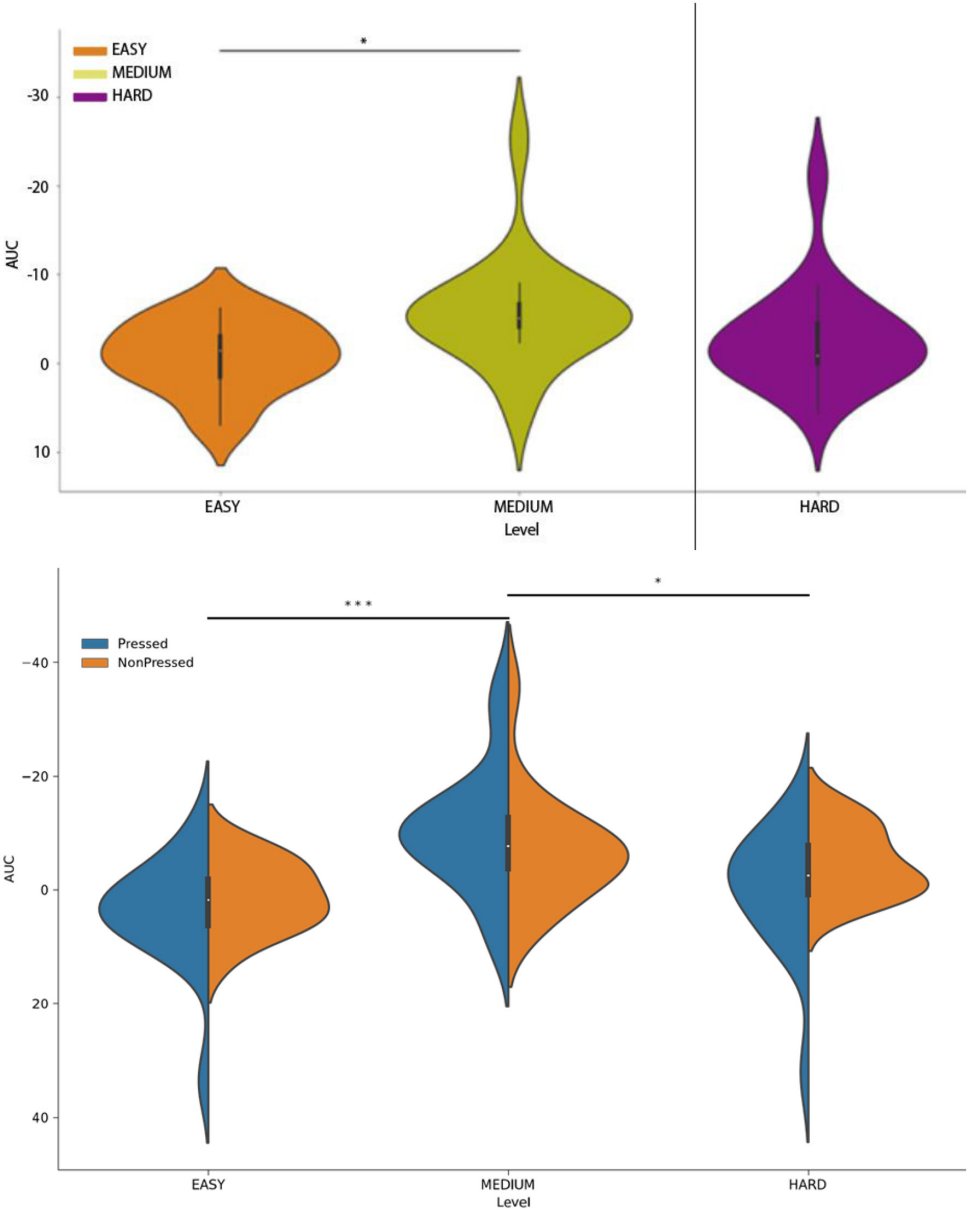
**(Upper panel)** Distribution of area-under-the-curve (AUC) values assessed separately for the press-locked time series in the easy trials (orange), medium trials (olive), and hard trials (violet). **(Lower panel)** Distribution of AUC values assessed separately for followed-by-press and press-free trials in the three experimental trial types. The solid line represents the time series terminated by a button press, while the dashed line represents the time series not followed by any button presses. All time series were derived from the Cz channel. *p < 0.05; ***p < 0.001.

The development of the RP waveform (Figure 4) is characterized by a clear, slow drift of the signal into the negative domain in the Cz electrode, which is a typical feature of the RP waveform (Shibasaki and Hallett, 2006). However, the onset of the potential differed across the three experimental conditions, as reflected in the variation in the distribution of the area-under-the-curve (AUC) values (Figure 7, upper panel). Specifically, an ANOVA with the factor *TrialType* indicated a difference in the AUC distribution between the experimental types (*F* = 2.1, *p* = 0.018). The pairwise comparison showed that AUC values differed specifically between the medium and easy trials (*F* = 4.3, *p* = 0.003).

When both followed-by-press and press-free trial-end-locked trials were included in the analysis (see Figure 4, lower panel), the difference in the distribution of AUC values persisted (*F* = 2.9, *p* = 0.024; factor *TrialType*). In this case, the AUC values differed specifically between the easy and medium trials (*F* = 2.7, *p* = 0.0006, zval = 3.39) and between the medium and control trials (*F* = 3.3, *p* = 0.007, zval = 2.67). Importantly, we did not observe any difference between the followed-by-press and press-free trials (*F* = 2.8, *p* = 0.013, zval = 2.1; factor *IsPressed*).

We additionally confirmed the similarity of the RP waveforms in the followed-by-press and press-free trials by plotting the end-of-trial-locked epochs (Figure 7). We showed that the standard error bars calculated for each time point in the time series overlapped between the followed-by-press and press-free trials in all three experimental conditions.

In addition, an assessment was conducted of the localization of the components of the readiness potential in relation to its lateralization. The activity of electrodes F5 and F6 (frontal area) was evaluated for this purpose. As illustrated in Figure 5, the pressing of a key evokes a specific activity. The dynamics associated with different decision conditions are visible. Figure 5 also illustrates the source analysis for the selected time period. For the “hard” condition, there is more involvement of the frontal and central regions of the cortex, as well as greater asymmetry.

## Discussion

In this study, we introduced a novel paradigm, C.R.A.B., which allowed us to compare the RP waveforms preceding movement in different experimental settings. Unlike simple self-paced movement tasks (Suwandjieff and Müller-Putz, 2024), our proposed framework allowed us to model the when-decision through indirect measurements without relying on the participants' reflective reports. At the same time, all mechanical parameters of the executed movements were identical.

As a proof-of-concept study, we piloted the paradigm on healthy adult participants and confirmed that the experimental settings induced different RP waveforms in various types of trials. In particular, we observed a difference between easy trials, during which the participants knew the exact location of the hider-crab from the beginning of the trial and could thus make a button-press decision immediately after the trial started, and medium trials, during which the participants could not be certain of the hider-crab's final location until the very end of the actor-crab's walk. Moreover, medium-difficulty trials explicitly required constant monitoring of the hider-crab's location in order to make the whether-decision (to press or not to press the button) and the what-decision (to press the left button or the right button), in accordance to the decision types outlined by (Brass and Haggard 2008).

Given that the RP waveforms were smaller (starting later and with less amplitude) in the condition where the hider-crab's final position was presented at the beginning of the trial (easy trials) than in the condition (medium and/or hard) characterized by evidence accumulation, our data support the evidence accumulation model (Schurger et al., 2012). According to this model, in easy trials, the presented evidence was strong enough to cross a relatively low threshold at the beginning of the trial (almost simultaneously with the trial onset), whereas in medium trials, the discrete-in-time presentation of evidence was insufficient to cross a relatively high threshold. We would also like to emphasize that in medium trails the main emphasis is on maintaining the subject's attention. Perhaps such a strong difference from other RP is related precisely to the control of the subject's attention. In this type of trail, the subject has to follow minimal changes on the screen until the end of the trial and make a decision at the very last moment.

Considering these results, it is interesting to speculate on the nature of RP waveform development in the game's hard trials. In our study, the hard trials did not provide any clues or hints regarding the hider's location. Essentially, this type of trial served as a control, as in the Libet paradigm (Libet et al., 1983). Previous studies have demonstrated the influence of various internal factors, such as breathing patterns (Park et al., 2008) and heartbeat cycles (Al et al., 2021), on participants' responses in the Libet paradigm. For example, research shows that specific breathing patterns reliably increase heart rate and improve decision-making (De Couck et al., 2019). These findings indicate that internal bodily processes play a role in decision-making, even when no explicit external cues are present. Therefore, it is plausible that in our study, the participants may have relied on some internal information, possibly related to bodily rhythms or other physiological cues, to guide their decision-making process in the hard conditions. In other words, this internal information could have served as evidence to be accumulated by the accumulator.

The absence of a difference between the followed-by-press and press-free trials can also be explained using the evidence accumulation model for the RP waveform (Schurger et al., 2012). The difference between followed-by-press and press-free trials in all trial types, except for the easy trials, can be explained by the ongoing accumulation of evidence, regardless of the final decision at the end of each trial. In easy trials, by contrast, no evidence accumulation occurred after the initial rapid input of evidence. If these explanations hold true, in future studies using the C.R.A.B. paradigm, we would expect to see differences in the point of no return (Schultze-Kraft et al., 2016) in vetoing movement at different stages of the experimental trial in medium and hard conditions. Given a presumably more prominent evidence accumulation in medium trials, we expect these differences to be stronger in the medium trials.

The discriminability of the RP waveforms across all types of experimental trials and their distinguishability from each other provide a solid ground for testing the paradigm in the child population. If the pilot results in adults are replicated, we suggest that the proposed framework could be useful for tracking the development of the RP waveform as a function of age. Additionally, it can be easily adapted for more decision-making scenarios, offering a means to disentangle the contribution of the decision-making process (as explained by the evidence accumulator model) from the purely motor pre-movement potential components described by (Shibasaki and Hallett 2006). Finally, we believe that this method could be useful in the design of brain-computer interfaces related to RP by identifying decision scenarios that elicit the most prominent RP patterns.

## Limitation

In our opinion, it is extremely difficult to create a paradigm in which all conditions can be reproduced and the “what,” “when,” and “whether” components can be separated. By adjusting the initial parameters of a given paradigm, one can only regulate the response to which the parameters and the design of the experiment are directed. For example, setting a time frame for decision-making limits the “spontaneity” of the “decision-making” process, but increases the role of attention. Changing the bonus points for different conditions controls the motivational contribution to making a particular decision. Therefore, it is impossible to “solve” the “Libet paradigm” using the same settings.

## Conclusion

In this study, we introduced a novel paradigm, C.R.A.B., to compare RP waveforms preceding movement in different experimental settings. Unlike simple self-paced movement tasks, the proposed approach allowed us to model the when-decision through indirect measurements without relying on the participants' introspective reports. At the same time, all the mechanistic parameters of the executed movements were identical.

In this proof-of-concept study, we piloted the framework on healthy adult participants and confirmed that the experimental settings induced different RP waveforms in different trial types. In particular, we observed a difference between easy trials, during which the participants knew the exact location of the hider-crab from the beginning of the trial and could thus make a button-press decision immediately after the trial started, and medium trials, during which the participants could not be certain of the hider-crab's final location until the end of the actor-crab's walk. Moreover, medium trials explicitly required constant monitoring of the hider-crab's location to make a whether-decision (to press or not to press) and a what-decision (to press the correct button).

The C.R.A.B. paradigm effectively demonstrated differences in RP waveforms across trial types, therefore, this paradigm can be used to study in detail the various components of the RP. Since this paradigm represents a game interface, it is possible for studying the RP in children, including children with various diseases. We believe that studying the change in the generation of RP at different stages of human development and with different disorders is an important component of assessing brain development.

## Generative AI statement

The author(s) declare that no Gen AI was used in the creation of this manuscript.

## Data Availability

The raw data supporting the conclusions of this article will be made available by the authors, without undue reservation.
