# Supplementary material for: C.R.A.B.: a gamified paradigm for studying readiness potential
Source: Front Hum Neurosci. 2025 Sep 3;19:1534412. doi: 10.3389/fnhum.2025.1534412 (PMC12442887; doi:10.3389/fnhum.2025.1534412)
Supplement: Supplementary file 1 [file Data_Sheet_1.pdf]

Table 1. The main articles related to the study of free will and their limitations.

**Authors**	**Title**	**Idea**	**Disadvantages of the method**
EEG methods			
Libet et.al. (1983)	The neural time—factor in perception, volition and free will	measure the moment of decision-making by spontaneously pressing a button	no control of attention level
Matsuhashi & Hallett (2008)	The timing of the conscious intention to move	Matsuhashi and Hallett introduced a clever method to catch intentions in the moment. They had participants make spontaneous movements but occasionally play a beep tone; if the person had already decided to move when the tone occurred, they were to veto the movement (The timing of the conscious intention to move). By varying tone timing and seeing when moves could be successfully vetoed, they inferred the timing of conscious intention without relying on memory.	no control of attention level
Schultze-Kraft et al. (2016)	The point of no return in vetoing self-initiated movements	Participants played a “duel” game trying to press a button, while a computer monitored their EEG. When the BCI detected the characteristic RP pattern, it would send a stop signal to try to halt the action.	a complex paradigm that uses BCI
fMRI			
Soon et al. (2008)	Unconscious Determinants of Free Decisions	Chun Siong Soon, John-Dylan Haynes and colleagues used fMRI to predict a person’s choice before they knew it themselves. Participants freely decided to press either a left or right button and noted when they became aware of their choice (similar to Libet, but with left/right decisions instead of just when to move).	difficult to control children attention in MRI scanner
Bode et al. (2011)	Ultra-High-Field fMRI Replication	To confirm and extend Soon et al.’s findings, Stefan Bode, Haynes and colleagues repeated the experiment with 7-Tesla fMRI (higher resolution) and multivariate decoding techniques.	difficult to control children attention in MRI scanner
Single-Neuron Recordings of Volition			
Fried et al. (2011)	Neuronal Prediction of Free Will	Itzhak Fried and colleagues recorded from hundreds of individual neurons in the medial frontal lobes of awake human patients who performed a Libet-style task. Patients had electrodes implanted for clinical reasons, giving a rare opportunity to probe deep brain structures.	invasive method
Psychological assessment (just example)			

Nadelhoffer T. et al.(2014)	The free will inventory: Measuring beliefs about agency and responsibility	5-item subscales designed to measure strength of belief in free will, determinism, and dualism. Part 2 consists of a series of fourteen statements designed to further explore the complex network of people's associated beliefs and attitudes about free will, determinism, choice, the soul, predictability, responsibility, and punishment.	there is no way to assess the exact moment of decision making

References:

Bode, S., He, A. H., Soon, C. S., Trampel, R., Turner, R., and Haynes, J. D. (2011). Tracking the Unconscious Generation of Free Decisions Using UItra-High Field fMRI. *PLoS One* 6, e21612. doi:10.1371/JOURNAL.PONE.0021612.

Fried, I., Mukamel, R., and Kreiman, G. (2011). Internally Generated Preactivation of Single Neurons in Human Medial Frontal Cortex Predicts Volition. *Neuron* 69, 548–562. doi:10.1016/j.neuron.2010.11.045.

Libet, B., Gleason, C. A., Wright, E. W., and Pearl, D. K. (1983). Time of conscious intention to act in relation to onset of cerebral activity (readiness-potential). The unconscious initiation of a freely voluntary act. *Brain* 106 (Pt 3), 623–642. doi:10.1093/BRAIN/106.3.623.

Matsuhashi, M., and Hallett, M. (2008). The timing of the conscious intention to move. *Eur. J. Neurosci.* 28, 2344–2351. doi:10.1111/J.1460-9568.2008.06525.X,.

Nadelhoffer, T., Shepard, J., Nahmias, E., Sripada, C., and Ross, L. T. (2014). The free will inventory: Measuring beliefs about agency and responsibility. *Conscious. Cogn.* 25, 27–41. doi:10.1016/j.concog.2014.01.006.

Schultze-Kraft, M., Birman, D., Rusconi, M., Allefeld, C., Görgen, K., Dähne, S., et al. (2016). The point of no return in vetoing self-initiated movements. *Proc. Natl. Acad. Sci. U. S. A.* 113, 1080–1085. doi:10.1073/PNAS.1513569112/SUPPL_FILE/PNAS.1513569112.SAPP.PDF.

Soon, C. S., Brass, M., Heinze, H. J., and Haynes, J. D. (2008). Unconscious determinants of free decisions in the human brain. *Nat. Neurosci.* 11, 543–545. doi:10.1038/NN.2112,.
